# Prognostic implications of MUC1 and XBP1 concordant expression in multiple myeloma: A retrospective study

**DOI:** 10.1371/journal.pone.0320934

**Published:** 2025-04-03

**Authors:** Sheerien Kareem Rajput, Khurram Minhas, Iqbal Azam, Sadia Habib, Usman Shaikh, El-Nasir Lalani

**Affiliations:** 1 Centre for Regenerative Medicine and Stem Cell Research, The Aga Khan University, Karachi, Pakistan; 2 Department of Pathology and Laboratory Medicine, The Aga Khan University, Karachi, Pakistan; 3 Department of Community Health Sciences, The Aga Khan University, Karachi, Pakistan; BMSCE: BMS College of Engineering, INDIA

## Abstract

Multiple myeloma (MM) is a disease of malignant plasma cells (PC) with poor survival. Disease progression and treatment relapse are attributed to MM cancer stem cells (CSCs) and signaling molecules such as MUC1 and XBP1. The study aimed to determine the prognostic value of expression of CSC-associated biomarkers, MUC1 and XBP1 in MM, which has not been explored previously. In this study, we determined the immunohistochemical expression of CSC markers (ALDH1, CD117, and CD34), MUC1, and XBP1 in 128 MM formalin-fixed paraffin-embedded bone marrow archival blocks. The expression of biomarkers was assessed for association with clinicopathological variables and patient survival. Descriptive analysis, survival plots and crude association between outcome and independent variables were assessed using Kaplan Meier and Log rank test. Univariate and multivariable analyses were performed using simple and multiple Cox regression models. The results are reported as crude and adjusted hazard ratios with 95% confidence intervals. Expression of ALDH1 and CD117 was found in 51% and 48% of the tumors, respectively. ALDH1 expression was associated with 1.83 years of reduced survival for patients with CD56-negative tumors. MUC1 expression was observed in 62%, whereas XBP1 was expressed in 48% of tumors. Combinatorial group analysis of XBP1 and MUC1 stratified patients into two prognostic groups. Cases with tumors negative for expression of MUC1 and XBP1 (XBP1-/ MUC1-) were categorized as a ***good prognostic group*** with increased survival of 3.42 years compared to cases with tumors expressing both (***Worst prognosis***, XBP1 + /MUC1+)**.** Concordant expression of MUC1 and XBP1 in MM defines a subset of patients with adverse outcomes. The adjusted hazard ratio showed a four-fold increased risk of mortality associated with the concordant expression of MUC1 and XBP1 in patients > 65 years of age.

## Introduction

Multiple myeloma is an incurable disease of neoplastic plasma cells that inhabit the bone marrow (BM) for growth and survival. Genetic and epigenetic factors contribute to molecular and phenotypic variations, leading to inter and intra-tumoral heterogeneity. Prognostication of MM patients is challenging as patients present with varied clinical symptoms, stages of the disease, and responses to treatment [1,2].

Over the last two decades, novel therapies have improved patients’ overall survival from a few months to over a decade in some cases [3]. However, despite this progress, MM remains an incurable chronic malignancy, and less than 10% of the patients experience long-term survival [4,5].

John Dick and Dominique Bonnet (1997) introduced the classical Stem cell model in the hematopoietic system. The model postulates that the whole mass of the cancerous cell population is dependent upon an undifferentiated, quiescent, and slow-proliferating group of cells known as the “Cancer Stem Cells” (CSCs) [6]. CSCs can recapitulate the original tumor along with multi-lineage differentiation potential, resistance to toxins, and unlimited proliferative capacity [7,8]. Identifying, isolating, and understanding this biologically distinct subpopulation of cells is essential for developing targeted and effective therapies. The first evidence for a quiescent and drug-resistant population in MM came from Drewinko and colleagues [9]. Although the genetically distinct type of MM CSCs markers’ expression and clinical relevance in patients with MM have not reached a consensus yet, independent or combinatorial expression of CD117, ALDH1, and CD34 are considered markers associated with the presence of MM-CSC-like cells.

MUC1 expression has been identified in 2–10% of normal human BM mononuclear cells, CD34 + cells, resting T cells, B cells, and plasma cells [10]. Aberrant expression of MUC1 has been demonstrated to play a seminal role in cancers, including MM [11–13]. MUC1-mediated nuclear translocation of β-catenin and NF-kB complex modulates tumor cell survival, anchorage-independent growth, and drug resistance in MM cells *in vitro* [14–16]. Bar-Natan and colleagues (2017) recently demonstrated that MUC1 expression was significantly up-regulated when MUC1-expressing MM cells were co-cultured with the HS5 stroma cell line. MM cells exhibited increased drug resistance and a high proliferative index in co-culture experiments. [17]. However, the prognostic value of MUC1 expression in predicting the outcome of patients with MM remains unclear [18,19].

X Box Binding Protein 1 (XBP1) is a basic-region leucine zipper (bZIP) protein belonging to the CREB/ATF (cAMP response element-binding protein/activating transcription factor) family of transcription factors (TF). XBP1 expression is indispensable for plasma cell differentiation and immunoglobulin synthesis. Significant evidence demonstrates the pathogenic role of XBP1 in cancers and its utility as a therapeutic target. Elevated levels of XBP1 have been shown to exhibit inverse prognostic significance in MM patients. Furthermore, high expression of XBP1 has been shown to correlate with advanced disease and poor prognosis in Thalidomide-treated MM patients [20,21]. The pathogenic role of XBP1 in MM was best described by Carrasco et al. (2007) when they induced XBP1 expression in transgenic mice, and all the mice developed MGUS resembling human disease. Also, within two years, 26% of the mice developed MM with increased secretion of monoclonal immunoglobulins [22]. Furthermore, XBP1-induced secretion of IL-6 supports the growth and survival of myeloma cells [23]. To our knowledge, clinical significance of XBP1 regarding treatment resistance and prognosis has not been reported to date.

There have been a handful of studies from Pakistan on MM and none examining the expression of CSC markers (CD34, CD117, and ALDH1), MUC1, and XBP1 and their potential association with prognosis and survival. This study evaluated the expression of selected markers in a cohort of MM patients and determined their prognostic relevance in terms of overall survival.

## Materials and methods

### Study design and sample collection

#### Ethical compliance.

This retrospective cohort study was conducted in complete adherence to the REMARK guidelines and the “Declaration of Helsinki” [24–26]. Ethical approval was obtained from the Ethical Review Committee of the Aga Khan University Hospital (AKUH) (reference number 2741-Pat-ERC-13).

#### Study setting and participants.

The study was undertaken at the Aga Khan University Hospital (AKUH), Pakistan. AKUH is a not-for-profit university teaching hospital receiving referrals from across Pakistan. The study included 128 treatment-naive patients diagnosed with symptomatic MM. Cases were identified through the MM registry maintained at AKUH and included diagnostic trephine biopsies of treatment naïve patients (S1 Fig). Between 2007 and 2014, 307 MM patients were diagnosed and registered at AKUH. Of these, 128 cases were selected based on the following criteria.

a) availability of formalin-fixed paraffin-embedded (FFPE) tissue blocks and
b) presence of representative tumor tissue on hematoxylin eosin-stained sections.

Prior to inclusion in the study, written informed consent was obtained from all study participants, granting permission to utilize their tumor tissues and associated data for research purposes.

### Data collection

This study adheres to stringent ethical guidelines, placing significant emphasis on safeguarding participants’ privacy and maintaining data confidentiality. The collected data underwent a rigorous anonymization process in which each sample was assigned numerical identifiers to conceal patient information effectively. This measure was implemented to comply with the REMARK guidelines and the policies set forth by AKU. Furthermore, it enhances the protection of participant identities to prevent unintended disclosure.

Archival Formalin-fixed paraffin-embedded (FFPE) trephine tissue blocks were retrieved from the Department of Pathology and Laboratory Medicine, AKUH, and medical records were reviewed. Clinicopathological data collected included gender, age at diagnosis, treatment regimens, ISS stage, and follow-up details. Data collection commenced on November 5, 2014, and encompassed the review of patients’ medical records, extending to November 24, 2020.

### Immunohistochemical expression

**Fig 1:**
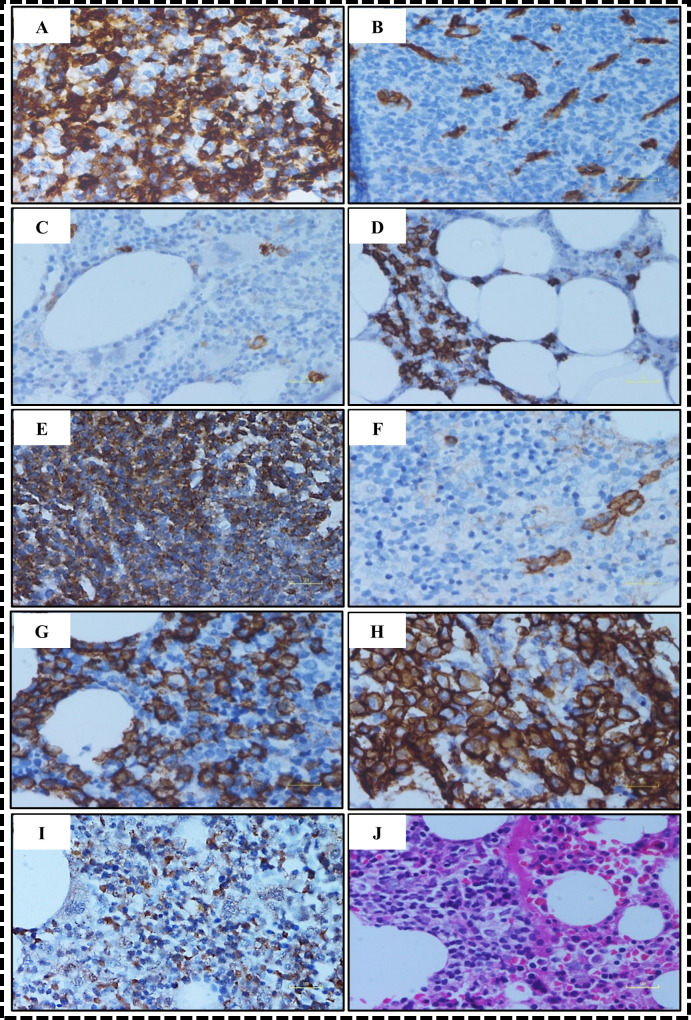
Photomicrographic collage showing expression of markers. Five-micron thick serial sections of FFPE trephine biopsies showing (A) Cytoplasmic expression of ALDH1, (B) Scattered tumor cells and endothelial cells (white arrow) showing membranous CD34 expression, (C) Isolated cells showing membrane expression of CD117, (D) cluster of tumor cells showing CD20 membranous expression, (E) Diffuse membranous expression of CD45, (F) Membrane expression of CD56, (G) Membrane expression of CD138, (H) Membranous and cytoplasmic expression of MUC1(EMA),(I) Weak nuclear and cytoplasmic expression of XBP1, (J) hematoxylin and eosin staining of trephine biopsy. Images were acquired through Olympus OlyVIA software, Magnification 20X.

**Fig 2:**
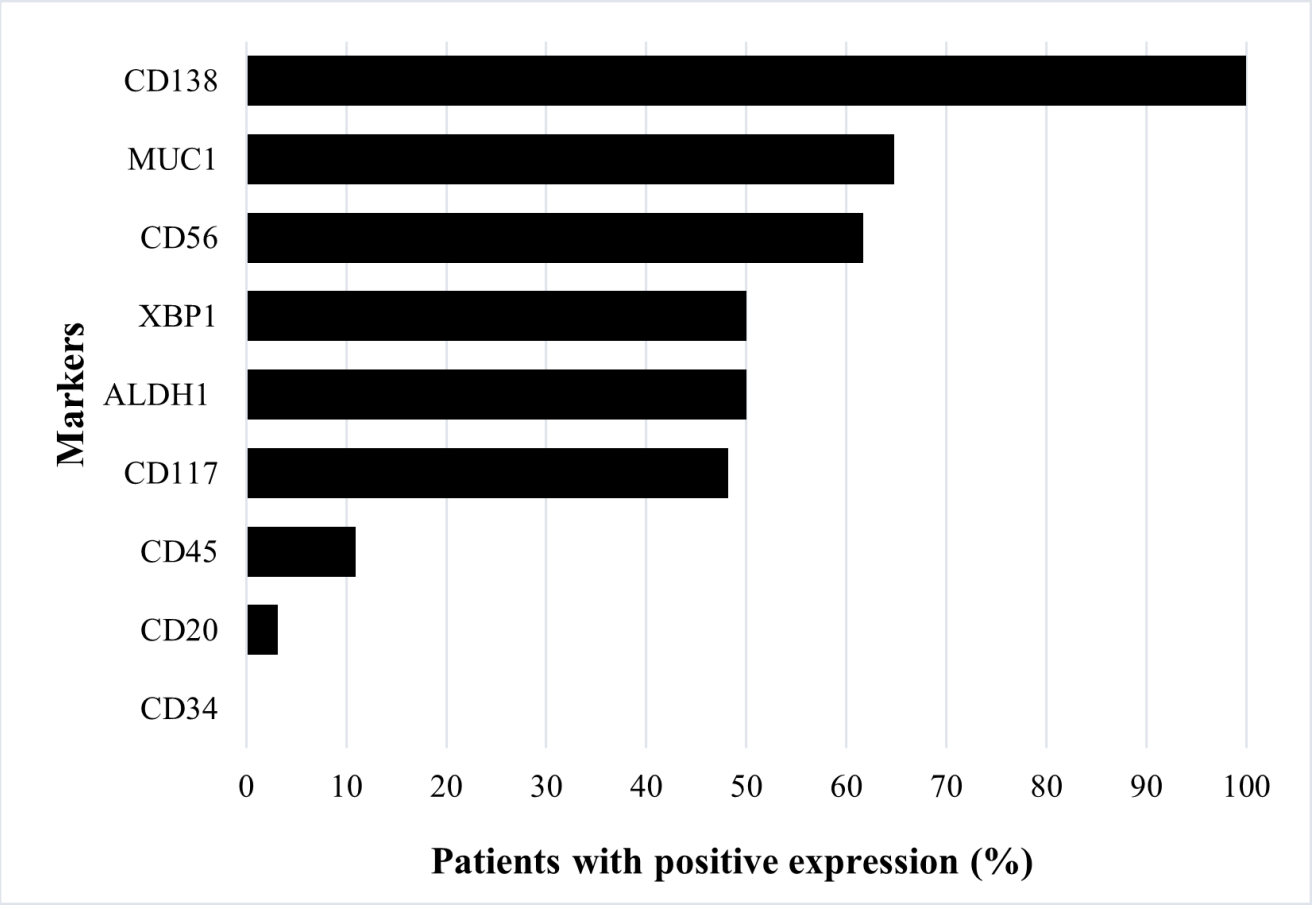
Distribution of expression of markers in FFPE trephine biopsies from MM patients.

Five-micron thick serial sections of FFPE trephine biopsies were cut and fixed onto poly-L-lysine coated slides to undertake IHC expression of ALDH1 (Fig 1A), CD34 (Fig 1B), CD117 (Fig 1C), CD20 (Fig 1D), CD45 (Fig 1E), CD56 (Fig 1F), CD138 (Fig 1G), MUC1 (Fig 1H) and XBP1 (Fig 1I). The details of antibodies used are described in S1 Table. The distribution of positive expression of markers in tumors is shown in Fig 2. Antibodies were diluted in EnVision^TM^ FLEX, Antibody Diluent (DM830). EnVision^TM^ FLEX, Mouse^,^ High pH (pH 9) kit by Dako (K8002) was used for tissue pretreatment and staining. Sections were dewaxed and processed for IHC as published previously [27]. Every fifth section was stained with hematoxylin and eosin to ensure the presence of representative tumor tissue (Fig 1J).

### Scoring for immunohistochemical expression

Slides were reviewed by consultant hematopathologists blinded to the patient’s clinical history. Blinding procedures were meticulously implemented throughout the slide evaluation process to mitigate potential biases. One pathologist, blinded to patient clinical history and other pertinent contextual information, independently reviewed each slide to assess the expression of the target biomarkers. Blinding was achieved by anonymizing patient identifiers and clinical details from the pathologist reviewing the slides. Meanwhile, the reliability and accuracy of the evaluations were ensured through analysis and verification by a second pathologist, unaware of the initial assessments. This involved independently re-evaluating a subset of the slides and cross-checking the expression levels determined by the first pathologist. Any discrepancies or uncertainties were resolved through consensus discussion between the two pathologists. We utilized CD138 expression to identify plasma cells (PC). IHC expression was evaluated, and plasma cells expressing the respective biomarkers were scored according to the criteria detailed in S2 Table [28].

### Statistical analysis

Data was entered and analyzed using Statistical Package for Social Sciences (SPSS) version 20. Descriptive statistics were computed as frequencies with percentages for the expression of immunophenotype markers and other variables. Medians with range are reported for quantitative variables such as age. Associations between the expression of markers and clinicopathological parameters were evaluated through the Chi^2^ test. The number of patients for each variable varies since we only had data for some factors for all study participants.

The date of the event; in case of death, and the date of the last follow-up were taken as an endpoint for patient survival analysis. The prognostic value of biomarkers was assessed for association with overall survival (OS) using the Log-Rank test, and Kaplan-Meier curves were plotted. Simple and multiple Cox regression for univariate and multivariable analyses was used to identify the risk of mortality associated with the expression of markers, and the resulting crude hazard ratios (HR) were reported with a 95% confidence interval. All the variables with a *p*-value of <  0.2 were considered eligible for multivariable analysis. Multivariable analysis using multiple Cox proportional hazard regression was calculated for individual markers after adjustment of other markers. The results were reported as adjusted hazard ratio with a 95% confidence interval, and a p-value of < 0.05 was considered significant unless mentioned otherwise. A global test of proportionality based on Schoenfeld residuals was used to assess proportionality hazard. Multicollinearity among eligible variables was also evaluated.

## Results

### Patient characteristics

The clinicopathological features of the patients are summarized in Table 1. Our study included 128 patients; of them, 88 were males. The mean age at diagnosis was 57 years (range 30–89 years), and 44% (n = 44/100) of the patients presented with ISS stage III disease. IgG myeloma was the most common immunoglobulin type. Almost 70% (n = 78/113) of the patients presented with bony lesions, while 51% of the patients (n = 52/101) had elevated levels of blood urea nitrogen (BUN) at the time of diagnosis. Impaired serum Beta-2 microglobulin was detected in 77.2% (n = 68/88) of the patients.

**Table 1:** Demographic characteristics of patients included in the study.

Variable	Frequency	%	Median (Range)
**Gender *(n = 128)***			
Female	**40**	**31.3**	
Male	**88**	**68.8**	
**Age at Diagnosis (years) (n = 128)**			
31-40	**9**	**7**	**57 (30-89)**
41-50	**24**	**18.8**	
51-60	**52**	**40.6**	
61-70	**25**	**19.5**	
71-80	**17**	**13.3**	
81-90	**1**	**0.8**	
**Serum Immunoglobulins (Heavy Chain)* (n = 118)***			
IgG	**63**	**53.3**	
IgA	**19**	**16.1**	
Light Chain Only	**21**	**17.7**	
Non-Secretory	**15**	**12.7**	
**Bone Lesions *(n = 113)***			
Absent	**35**	**31**	
Present	**78**	**69**	
**BUN *(n = 101)***			
Normal (6–24 mg/dL)	**49**	**48.5**	**33 (7-118)**
High (MALE > 24, FEMALE > 21)	**52**	**51.4**	
**Calcium Level *(n = 113)***			
Normal (8.6–10.2 mg/L)	**79**	**69.9**	
Low (<8 mg/L)	**23**	**20.3**	**9 (5.3-15)**
High (>11 mg/L)	**11**	**9.7**	
**Serum Creatinine *(n = 117)***			
Normal (0.8–1.3 mg/L)	**51**	**43.5**	**1.3 (0.30-9.6)**
Low (<0.8 mg/L)	**12**	**10.2**	
High (>1.3 mg/L)	**54**	**46.1**	
**Serum Beta-2 Microglobulin (n = 88)**			
Normal (1–3 mg/L)	**20**	**22.7**	**5.5 (0.33-47)**
High (>3 mg/L)	**68**	**77.2**	
**Serum Albumin *(n = 95)***			
Normal (3.5–5.2 mg/L)	**35**	**36.8**	**3.1 (1-5.1)**
Low (<3 mg/L)	**60**	**63.1**	
**ISS Stage *(n = 100)***			
Stage 1	**35**	**35**	
Stage 2	**21**	**21**	
Stage 3	**44**	**44**	

Abbreviations: BUN (Blood Urea Nitrogen), Ig (Immunoglobulin), ISS (International Staging System).

Only 17 cases of the study population were tested for chromosomal aberrations at diagnosis. Of these, hyperploidy with complex karyotypes was observed in only three cases, while 59% (10) displayed normal karyotypes by conventional G-banding karyotyping. Fluorescence *in situ* hybridization (FISH) analysis was undertaken to detect *IGH* (14q32) translocation, *TP*-*53* deletion (17p), and 13q deletion in 6, 7, and 8 patients, respectively. None of these cases showed the presence of the tested abnormalities.

The mean duration of follow-up was three years (SD ± 2.5 years), and 39 deaths were recorded in the entire cohort. During the study period, 79% (n =  87/110) of the participants reported relapse at least once.

### Association of clinicopathological parameters with survival

**Fig 3:**
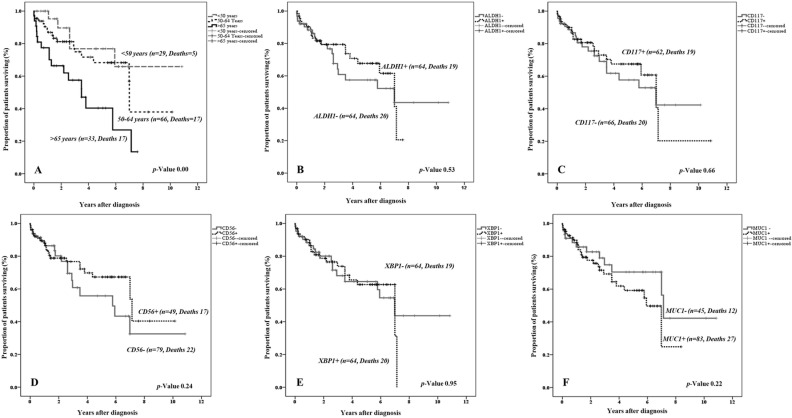
Kaplan Meier Curve showing overall survival of patients. The Log-rank test was used to assess the prognostic significance of clinicopathological features and expression of biomarkers. The Kaplan-Meier curve was used to estimate survival probabilities with 95% confidence intervals. The graphs show survival of patients segregated through: (A) Age *(p-value < 0.001),* (B) expression of ALDH1 *(p-value > 0.001)*, (C) expression of CD117 *(p-value > 0.001)*, (D) expression of CD56 *(p-value >  0.001)*, (E) expression of XBP1 *(p-value > 0.001)* and, (F) expression of MUC1 *(p-value > 0.001)*.

Amongst clinicopathological parameters, a significant association was observed between age groups and OS ***(p-value <  0.01)***. Patients were divided into three age groups: < 50 years, 50–64 years, and > 65 years. In the three age groups, the mean survival time was 8.27 years (95% CI 6.38–10.16), 6.56 years (95% CI 5.15–7.97), and 3.76 years (95% CI 2.63–4.88), respectively ***(p-value <  0.01).*** The univariate Cox proportional hazard analysis showed a threefold increased risk of mortality associated with advanced age (HR =  3.70, 95% CI-1.36–10.10) (see Fig 3A and S3 Table).

### Expression of markers and their association with clinicopathological parameters and survival

The Chi2 test was used to examine the relationship between the expression of markers and clinicopathological characteristics. Their corresponding p-values were derived by pairwise statistical analysis. The Log-rank test was used to assess the prognostic significance of biomarkers, and the Kaplan-Meier curve was used to estimate survival probabilities, with 95% confidence intervals.

None of the tumors showed CD34 expression, while CD20 was expressed in 3.97% of the cases. Hence, CD34 and CD20 were excluded from further statistical analysis. All the hazards were found to be proportional for all the variables used in this study when tested for the global test of proportionality based on Schoenfeld residuals (see Table 2).

**Table 2:** Univariate and multivariable analysis for association of clinicopathological, demographic factors and expression of biomarkers with overall survival (OS) or hazard of mortality among MM patients (n = 128).

Prognostic Factors	Crude Hazard Ratio (HR) (95% CI)	*p-value*	Adjusted Hazard Ratio (AHR) (95% CI)
**Gender**		***0.52***	
**Female**	1		
**Male**	1.25 (0.62-2.53)		
**Age at Diagnosis**		***<0.001***	
**<50**	1		1
**50-64**	1.56 (0.57-4.25)		1.59 (0.58-4.35)
**>65**	3.70 (1.36-10.10)		4.10 (1.49-11.27)
**ALDH1**		**0.54**	
**Negative**	1		
**Positive**	0.82 (0.43-1.54)		
**CD117**		***0.66***	
**Negative**	1		
**Positive**	0.87 (0.46-1.63)		
**CD45**		***0.81***	
**Negative**	1		
**Positive**	1.11 (0.43-2.82)		
**CD56**		***0.25***	
**Negative**	1		
**Positive**	0.68 (0.36-1.30)		
**MUC1**		***0.22***	
**Negative**	1		
**Positive**	1.54 (0.76-3.13)		1.77 (0.85-3.60)
**XBP1**		***0.95***	
**Negative**	1		
**Positive**	1.01 (0.53-1.91)		1.06 (0.54-2.05)

#### ALDH1.

Cytoplasmic expression of ALDH1 was observed in 50% of the cases. However, it was not associated with the overall survival (OS) when patients were stratified based on ALDH1 expression in tumors (ALDH1 + (mean survival 5.42 years, HR = 0.82, 95% CI-0.43–1.54)) ***(p-value >  0.05)*** (Fig 3B).

A significant association was identified between ALDH1 expression and patients diagnosed at an advanced age (>57 years of age). Sixty-five percent of the tumors in this age group expressed ALDH1 ***(p-value <  0.05)***. Significant associations were also identified between concordant expressions of ALDH1 with CD117 ***(p-value <  0.01)*** and CD56 ***(p-value <  0.01)***; however, it did not yield prognostic information in terms of OS or clinicopathological variables.

#### CD117.

Membrane expression of CD117 was found in 48% of the cases and was associated with a 23% lower risk of mortality (mean survival six years, HR =  0.87, 95% CI- 0.46–1.63) ***(p-value >  0.05)*** (See Fig 3C). CD117 expression showed significant associations with CD56 ***(p-value <  0.05)*** and ALDH1 ***(p-value < 0.001)*** expressing tumors. There were no significant differences in clinicopathological variables between tumors with and without CD117 expression ***(P >  0.05)***.

#### CD56.

Membrane expression of CD56 was detected in 62% of the patients, which was associated with a 32% reduced risk of mortality (mean survival 6.5 years, HR =  0.68, 95% CI – 0.36–1.30) when compared to cases lacking CD56 expression ***(P >  0.05)*** (Fig 3D). Significant concordance was observed in expression of CD56, CD117 ***(p-value <  0.05)***, XBP1***(p-value <  0.05)*** and ALDH1 ***(p-value < 0.001)***. There were no significant differences in clinicopathological variables between tumors with and without CD56 expression ***(P >  0.05)***.

#### XBP1.

Cytoplasmic and nuclear expression of XBP1 was observed in 50% of the cases. Significant concordance was observed between expressions of XBP1 with CD 117 ***(p-value < 0.001)*** and CD56 ***(p-value <  0.05)*** expression. We did not find any associations of XBP1 expression with ‘patients’ survival and clinicopathological variables ***(p-value >  0.05)*** (Fig 3E).

#### MUC1.

Sixty-five percent of the cases showed membranous and cytoplasmic expression of MUC1 in tumors. Cases stratified based on MUC1 expression did not show any associations with clinicopathological parameters and expression of other biomarkers. However, a survival advantage of 1.9 years was found in cases lacking MUC1 expression in their tumors (MUC1 + tumors; mean survival 5.15 years, MUC1- tumors; mean survival 7.05 years, HR =  1.54, 95% CI – 0.76–3.13)) ***(p-value >  0.05)*** (Fig 3F).

### Association of combinatorial groups with survival

Combinatorial groups were analyzed to explore further the prognostic value of concordant expression of markers using the Chi^2^ test.

### ALDH1 & CD56; Combinatorial group

Cases were categorized into four groups based on ALDH1 and CD56 expression. OS analysis delineated these combinations into four prognostic groups.

- *Good Prognosis: ALDH1-/CD56 + , mean survival 6.57 ±  0.96 years (*n = 32)
- *Intermediate Prognosis ALDH1-/CD56-*, *mean survival 6.17 ±  0.96 years* (n = 32)
- *Poor Prognosis: ALDH1 + /CD56 + , mean survival 5.72 ±  0.41 years,* (n = 32)
- *Worst Prognosis: ALDH1 + /CD56-, mean survival 3.89 ±  0.76 years* (n = 17)

**Fig 4:**
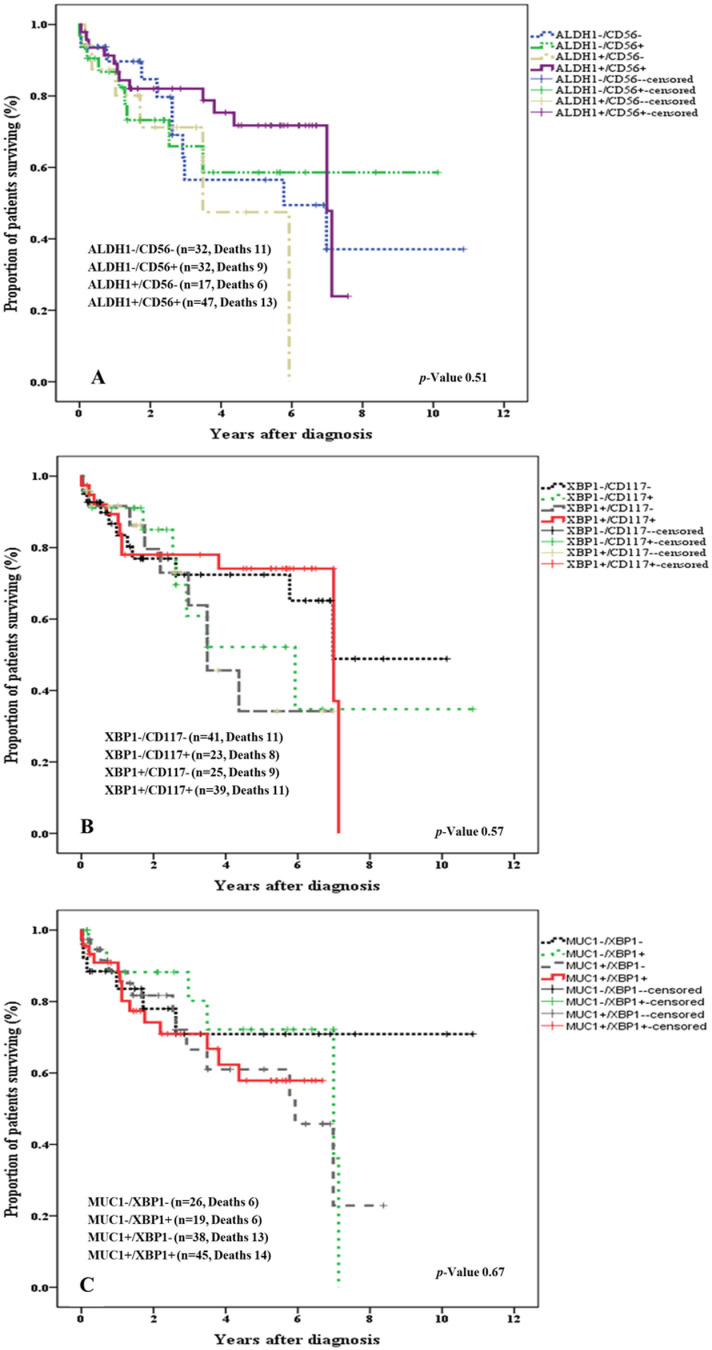
Kaplan Meier Curve showing OS of MM patients with tumors showing: The Log-rank test was used to assess the prognostic significance of the combinatorial expression of biomarkers. The Kaplan-Meier curve was used to estimate survival probabilities with 95% confidence intervals. The graphs show survival of patients segregated through: (A)combinatorial expression of ALDH1 and CD56, (B) combinatorial expression of XBP1 and CD117, and (C) Combinatorial expression of MUC1 and XBP1.

Combinatorial group analysis corroborated the protective effect of CD56 expression. Patients with tumors expressing ALDH1 but lacking CD56 expression (*ALDH1 + /CD56-)* had the worst prognosis, with a mean OS of 3.89 years. In comparison, the expression of CD56 (*ALDH1 + /CD56+)* showed a survival advantage of 1.82 years with a mean OS of 5.72 years ***(p-value >  0.05)*** (Fig 4A).

### XBP1 & CD117; Combinatorial group

Combinatorial group analysis with XBP1 and CD117 resulted in the following four prognostic groups:

- *Good Prognosis: XBP1-/CD117-, mean survival 6.78 ±  0.81 years (*n = 41)
- *Intermediate Prognosis: XBP1-/CD117 + , mean survival 5.87 ±  1.17 years (*n = 23)
- *Poor Prognosis: XBP1 + /CD117 + , mean survival 5.54 ±  0.46 years (*n = 39)
- *Worst Prognosis: XBP1 + /CD117-, mean survival 4.14 ±  0.61 years (*n = 25)

Subgroup analysis of XBP1 and CD117 revealed the favorable prognostic effect of CD117 expression and the adverse prognostic effect associated with XBP1 expression. Amongst cases lacking CD117 expression, patients with XBP1 expressing tumors showed 2.64 years reduced survival (*XBP1 + /CD117-, mean survival 4.14 ±  0.61 years)* compared to patients with XBP1 negative tumors (*XBP1-/CD117-, mean survival 6.78 ±  0.81 years)* ***(p-value >  0.05)*** (Fig 4B).

### MUC1 & XBP1; Combinatorial group

Independent expression of MUC1 and XBP1 showed a trend towards inferior OS. The prognostic value of MUC1 and XBP1 concordant expression was investigated in combinatorial groups. Subgroup analysis delineated into four distinct groups:

- *Good prognosis (MUC1-/XBP1-) mean survival of 8.02 ±  0.99 years (*n = 26)
- *Intermediate prognosis (MUC1-/ XBP1 + /) mean survival of 5.66 ±  0.66 years (*n = 19)
- *Poor prognosis (MUC1 + / XBP1-) mean survival of 5.14 ±  0.60 years (*n = 38)
- *Worst prognosis (MUC1 + / XBP1+) mean survival time of 4.6 ±  0.431 years (*n = 45)

Kaplan Meier’s analysis revealed that concordant expression of MUC1 and XBP1 increased mortality risk. Though independent expression of either MUC1 or XBP1 [(XBP1 + / MUC1-) & (XBP1-/ MUC1+)] showed increased survival advantage, patients whose tumors expressed both (XBP1 + / MUC1+) had a 3.42-year reduced survival. Only 50% of the patients with XBP1 + /MUC1 + tumors survived for four years compared to 70% of patients who survived for > 10 years when tumors lacked concordant expression of XBP1 and MUC1 ***(p-value >  0.05)*** (Fig 4C).

### Multivariable analysis using multiple Cox regression models for OS

We undertook multivariable analysis to examine the adjusted hazard ratios (AHR) associated with different variables. XBP1 was included in the multivariable analysis to evaluate its biological relevance in the presence of MUC1. We found poor prognosis associated with advanced age at the time of diagnosis (>65 years) (adjusted HR = 4.10, 95% CI- 1.49–11.27) ***(p-value < 0.001)*** and the risk of mortality increased up to four folds in patients whose tumors expressed MUC1 (adjusted HR = 1.77, 95% CI-0.85–3.60) ***(p-value >  0.01)*** and XBP1 (adjusted HR = 1.06, 95% CI- 0.53–2.05) ***(p-value >  0.01)*** (Table 2).

## Discussion

To our knowledge, this is the first study from Pakistan that describes the expression and prognostic significance of stem cell markers, lineage markers, MUC1, and XBP1 in BM trephines from MM patients. The salient findings from this study are as follows:

1. MM patients in Pakistan present approximately a decade earlier than in Western countries.
2. A high percentage of patients had tumors expressing stem cell-associated markers (CD117 and ALDH1) compared to the data from the rest of the world. However, the prognostic significance of this association could not be established.
3. Combinatorial group analysis revealed the adverse prognostic value associated with ALDH1, XBP1, MUC1 and their concordant expression.

### MM patients presented a decade earlier in Pakistan

MM is considered a rare malignancy diagnosed between the ages of 65 and 74 years with a male preponderance [5,29,30]. In our study, the patients were diagnosed at least a decade earlier (median age 57, range 30–89 years) compared to 70 years in the Western countries [31]. The SEER (Surveillance, Epidemiology, and End Results) data on U.S. incidence rates by age at diagnosis indicates an increase in incidence with age, from 8.5 per 100000 individuals (aged 50–54 years) to 51.8 per 100 000 individuals (aged 80–84 years) [32].

Early-onset MM has previously been reported in Pakistan [33–37] and other Asian countries, including India [38,39], Bangladesh [40], and China [41]. However, the differences between average life expectancies should be considered when comparing this data. Kim, K et al. studied and compared the clinical features of MM patients in the Asian population between the 1960s and 2010 and discovered a link between population life expectancy and the median age at MM diagnosis. They found that an increase in average life expectancy in the Korean population from 64 years in 1960–81 years in 2010 was associated with a rise in the median age at which MM patients presented from 54 to 67 years. They proposed that exposure to chemical carcinogens, ionizing radiation, air pollution, Westernized diets, and obesity were factors in their study population’s high MM incidence. [42].

The average life expectancy in Pakistan is ~  67 years compared to ~ 77 and 81 years in the United States and the United Kingdom, respectively [43]. Global life expectancy is projected to increase from 71 in 2022–81 in 2098 [44]. It is conceivable that the age at which patients present will shift to a higher age band, reflecting trends in Europe, America, Korea, and other countries. If the current lower age at presentation persists, it may prompt researchers to investigate the link between the early onset of MM and modifiable risk factors.

### ALDH1 is associated with an adverse prognosis

Aldehyde dehydrogenases (ALDHs) are a group of nicotinamide-adenine dinucleotide phosphate–positive (NAD(P)+) -dependent enzymes involved in retinoic acid metabolism and detoxification in cells. In our study, ALDH1 expression was found in 50% of the tumors, which is higher than reported in other MM studies [45]. High expression of ALDHs has been found in neural [46], prostate [47], colon [48], and breast [45,49] tissues and cancers previously.

The univariable analysis did not reveal any associations between ALDH1 and OS. However, combinatorial group analysis demonstrated adverse outcomes associated with ALDH1 expression in tumors lacking CD56 expression. Amongst patients with CD56 negative tumors, ALDH1 expression in tumors (ALDH1 + /CD56-) showed 2.28 years of inferior survival, suggesting that CD56 expression abrogates the adverse effect of ALDH1.

ALDHs govern drug resistance, activation of BCL2, AKT, drug efflux pumps and differentiation in cancer stem cells [50,51]. ALDH1 expression has been used to identify CSC-like populations in epithelial and hematological malignancies, including MM, and has been associated with drug resistance and relapse [52,53]. Recently, Tripathi et al. reported that MM heparanase high cells promoted stemness, including spheroid formation in the CAG MM cell line. Heparanase increased the expression of GL1I, SOX2 and ALDH1A, the predominant form of ALDH1 in MM [54]. Yang et al. showed that overexpression of ALDH1A1 increased resistance to doxorubicin and bortezomib *in vivo* and *in vitro* [55]. We were unable to investigate the relationship between ALDH1 expression and increased drug resistance due to incomplete follow-up and treatment regimen variability. However, immature morphological features were observed in CD138-expressing plasma cells. Low (4%) expression of CD20 in these cases further corroborates the finding. Moreover, a statistically significant association was also found between the concordant expression of ALDH1 and CD117.

Since half of the patients in our study expressed ALDH1, inhibition of ALDH1 expression could be considered a therapeutic approach for drug-resistant MM. However, this warrants larger cohort studies in ethnically diverse populations with complete clinical information followed by *in vitro* and animal model studies.

### CD56 expression and prognosis

CD56 is a neural cell adhesion molecule (NCAM) and a member of the immunoglobulin superfamily that is expressed in developing and mature cells of the human nervous system and natural killer (NK) cells. It is expressed in 70–80% of cases of MM but not in normal plasma cells [56].

CD56 expression was observed in 60% of our cases, which is comparable to previous reports (see Table 3). In our study, 70% of the tumors of patients over the age of 65 years exhibited CD56 expression ***(p-value <  0.05)***. Combinatorial group analysis showed that CD56 expression abrogated the adverse prognostic impact of ALDH1. Amongst patients with ALDH1 expressing tumors, expression of CD56 contributed to a survival advantage of 1.83 years (mean survival 5.72 years). It was also found to be associated with CD117, another marker of CSC-like cells. The positive outcome associated with CD56 expression without CD117 was confirmed by combinatorial group analysis.

**Table 3:** Expression of biomarkers (%) in the studied cohort and their comparison with published studies.

Marker		Expression Frequency in Tumors	
**Current Study (%)**		**Rest of the world (%)**	**Reference**
**CD34**	0	0	(Kimlinger & Witzig, 1997)
**ALDH1**	51	19-30	(Ginestier et al., 2007)
**CD117**	48	32	(Mateo et al., 2008; Pan et al., 2016)
**CD20**	4	6- 49	(Robillard et al., 2003; Mateo et al., 2008; Yavasoglu et al., 2015; Pan et al., 2016)
**CD45**	10.6	27, 73	(Santra, Shaughnessy Jr, & Bellamy, 2011; Flores-Montero et al., 2016)
**CD56**	60.3	60-74	(Guo et al., 2016; Pan et al., 2016)
**MUC1**	62.3	55-60	(Baldus, Palmen, & Thiele, 2007; Andrulis et al., 2014)
**XBP1**	48.3	70	(Carrasco et al., 2007)

Recent work by Koumpis et al. supports our observation as they found that lack of CD56 expression in MM patients was associated with clinical presentation with elevated levels of lactate dehydrogenase (LDH) and β2-microglobulin. However, it was not associated with patient survival [57].

Taouk and colleagues recently demonstrated the utility of CD56 as a predictor of NK-mediated cytotoxicity in breast carcinoma cell lines. CD56 enhanced NK-mediated cytotoxicity by facilitating the interaction between NK and malignant cells, which led to cytotoxic enzyme transfer to NK cells, inducing Caspase-3-mediated activation in the target cell [58]. It is conceivable that CD56 expression in myeloma cells activated the NK-mediated cytotoxic immune response, leading to cancer cell death, thereby contributing to a favorable prognosis.

### CD117 expression and prognosis

CD117, commonly known as c-kit or stem cell factor receptor, is a “KIT-proto-oncogene receptor tyrosine kinases” expressed in non-malignant cells, including hematopoietic stem cells (HSCs), melanocytes, mast cells, and breast epithelium, but not on plasma cells, gastrointestinal stromal tumors, acute leukemias, myelodysplastic syndromes, monoclonal gammopathies and MM. In our study, CD117 expression was observed in 48% of the cases compared to 32% reported in the literature [59,60]. Expression of CD117 was associated with ALDH1 ***(p-value 0.005)***, CD56 ***(p-value 0.01)***, and XBP1 ***(p-value 0.005)*** expression. When CD117 expression was examined for potential association with five-year survival, it did not correlate with prognosis independently or in combination with ALDH1, CD56, and XBP1.

Although CD117 plays a vital role in cell signaling, survival, proliferation, migration, and cellular differentiation [61], no consensus has yet been established regarding the prognostic significance of CD117 expression in MM [3,62].

### XBP1 expression and prognosis

XBP1 signaling pathway plays a vital role in the pathogenesis of MM. XBP1 is an essential mediator of the unfolded protein response (UPR) and a key component in developing fetal hepatocytes, acinar cells, and plasma cells [63]. It acts as a multifunctional transcription factor (TF).

XBP1 expression did not show any associations with clinicopathological parameters in this study. Lack of XBP1 expression in tumors showed a survival advantage of 1.46 years compared to XBP1 +  tumors. However, it did not reach statistical significance. The inverse prognostic value of XBP1 expression was further substantiated through XBP1/ CD117 combinatorial group analysis, which revealed a 2.64-year survival advantage associated with lack of XBP1 expression in CD117-negative tumors.

There is growing evidence that XBP1 plays a significant role in cancer pathogenesis and can be used as a therapeutic target. *In vitro* studies have shown that XBP1overexpression promotes proliferation and drug resistance in MM [20,21,64]. The pathogenic role of XBP1 in myeloma was best described by Carrasco et al. (2007) when they induced XBP1 expression in transgenic mice and discovered that all the mice developed MGUS, resembling human disease. Furthermore, 26% of the mice developed MM with increased monoclonal immunoglobulin secretion within two years [22]. Previously, XBP1 expression has been linked to poor outcomes in BCa [65], B-cell lymphomas [66], and osteosarcomas [67]. However, the prognostic significance of XBP1 in MM is still bipartisan. Bagratuni et al. demonstrated that a high XBP1 spliced/ unspliced ratio of mRNA is an independent prognostic indicator associated with poor survival in patients treated with thalidomide [21]. While Gambella et al. have shown that high XBP1 mRNA expression is associated with improved overall survival in patients treated with bortezomib [68]. Lee and colleagues have demonstrated that inhibiting the IRE1-XBP1 pathway in MM cells disrupts the activation of the spliced form of XBP1 and stabilizes the unspliced form, acting as a double negative, thereby sensitizing the cells to ER stress-induced apoptosis [64].

Further large cohort studies are required to determine the prognostic value of XBP1. Moreover, its association with a stem cell marker, CD117, needs further functional evidence. Since XBP1 is a mediator of the UPR, prospective studies can be designed to study the changes in XBP1 expression and its role in MM onset in MGUS patients.

### MUC1 expression and prognosis

MUC1 is a multifaceted protein playing a seminal role in tumorigenesis and its expression has been linked to poor overall and disease-free survival in carcinomas of the breast, prostate, lung, stomach, and ovary [69–71]. Hematopoietic cells express MUC1 to a lesser extent, but its aberrant expression has been reported in hematological malignancies. MUC1 expression was found to be associated with a trend toward adverse outcomes in this study. Patients with tumors lacking MUC1 expression had a 2-year survival advantage over patients with tumors expressing MUC1.

*In vitro* studies in MM have demonstrated that MUC1 expression is associated with a CSC-like phenotype, drug resistance, and proliferation [11–13,16,17,72]. This would suggest that *in vivo* MUC1 expression may translate to an adverse outcome. Recently, the adverse prognostic effect of MUC1 expression has been reported in acute myeloid leukemia (AML) [73]. However, its prognostic significance in MM has not been reported. The reason studies do not demonstrate this adequately is due in part to the limited repertoire of antibodies to epitopes on different regions of MUC1: glycosylated, partially glycosylated, unglycosylated extracellular domain and to epitopes on the highly conserved cytoplasmic domain.

MUC1 is considered an attractive target for therapeutic intervention due to its differential expression patterns and glycosylation on normal and malignant cells. However, this variability in expression and glycosylation patterns contributes to inconsistencies in detecting MUC1 in diverse cancer types and within different cases of the same disease. The following factors should be considered while studying MUC1 expression:

1. Different components of MUC1 protein exhibit differential oncogenic activities. Expression of the secreted form of MUC1, also known as soluble MUC1 (sMUC1), is associated with tumor burden and can be effectively targeted using the B27.29 antibody for CA27.29 epitope present on sMUC1 [74]. Likewise, the MUC1 Ab-5 antibody recognizes the epitope present at the cytoplasmic tail (CT) at the carboxylic end of the protein. It provides significant information regarding the signaling functions of the protein and its association with stem cells [12]. Hence, the expression of B27.29 may not yield information regarding the signaling molecules, and MUC1 Ab-5 may not render any association with patient survival.
2. MUC1 Extracellular domain (ECD) contains Variable Number of Tandem Repeats (VNTR), which consists of variable repeats of 20 aa. In healthy tissues, five potential sites within the VNTR domain are heavily glycosylated; however, overexpression of MUC1 during neoplastic transformation alters the glycosylation pattern of these sites, and tumor cells may express aberrantly glycosylated MUC1. Sugar moieties have been implicated in antibody recognition and binding of MUC1 antibodies developed against VNTR domain epitopes [75,76]. As a result, the patterns and degree of glycosylation may influence antibody-based protein detection.
3. High polymorphism of MUC1 protein is attributed to the VNTR domain. It has been demonstrated that variable amino acid sequences with varying numbers of repeats can exist in different cell lines. Furthermore, increased length of the VNTR domain may also result in poor antibody penetration and immunogen unavailability [77,78]. Hence, studies evaluating the prognostic significance of MUC1 are inconclusive and partial, depending upon the various factors discussed above.

Analysis of MUC1 and XBP1 concordant expression further corroborated the adverse outcome observed with MUC1 expression. We found that in a small cohort of tumors negative for XBP1 and MUC1 expression contributed to a 3-year survival benefit with a mean survival of 8.02 years compared to a mean survival of 5.14 years in MUC1expressing tumors. Concordant expression of MUC1 and XBP1 exerted a modifier effect on adverse outcomes with a means survival of 4.6 years. Multivariable Cox regression analysis showed that the expression of MUC1 and XBP1 in elderly patients (>65 years) increased mortality risk up to 4 times compared to other age groups. Proteins rarely act as isolated molecules in system biology and over 80% of the known proteins operate in complexes [79]. The observation of an association between MUC1 and XBP1 and its adverse association with outcome is intriguing and raises the possibility of a functional link or crosstalk between them.

A higher prevalence (11%) of Non-secretory Multiple Myeloma (NSMM) was identified in this study, which was similar to a recent report from China, where 10.58% of non-secretary MM cases were reported [80]. NSMM has been reported as a rare variant, representing 3–5% of MM patients. Recently, NSMM has been redefined and subclassified into four categories: a) oligo-secretors, b) non-producers, c) true non-secretors and d) false non-secretors [81,82]. It is conceivable that reanalyzing our cases with more sensitive techniques and re-categorizing them according to the new criteria of NSMM may lower the prevalence of true NSMM in this study.

This is a single-center retrospective study. The minor variations in the results can be attributed to a) fixation protocols, b) antibodies used, c) scoring methods and d) sample size. Major challenges encountered during our study were incomplete follow-up and a relatively small sample size. Furthermore, because treatment strategies were not uniform, our study cohort was a mixed group in terms of treatment regimens. Inclusion of patients who received uniform treatment would have helped to clarify the relationship between these markers and patient survival in different treatment groups.

## Supporting information

S1 Fig Experimental plan and sampling for IHC. (TIF)

S1 Table Panel of antibodies used for immunohistochemical expression in FFPE tissues of myeloma patients. (DOCX)

S2 Table Scoring criteria for IHC expression [28]. (DOCX)

S3 Table Survival analysis for association of ‘biomarkers’ expression with overall survival in patients with MM (n = 128). (DOCX)
