# Supplementary figures and images for: Prognostic implications of MUC1 and XBP1 concordant expression in multiple myeloma: A retrospective study
Source: PLoS One. 2025 Apr 3;20(4):e0320934. doi: 10.1371/journal.pone.0320934 (PMC11967961; doi:10.1371/journal.pone.0320934)

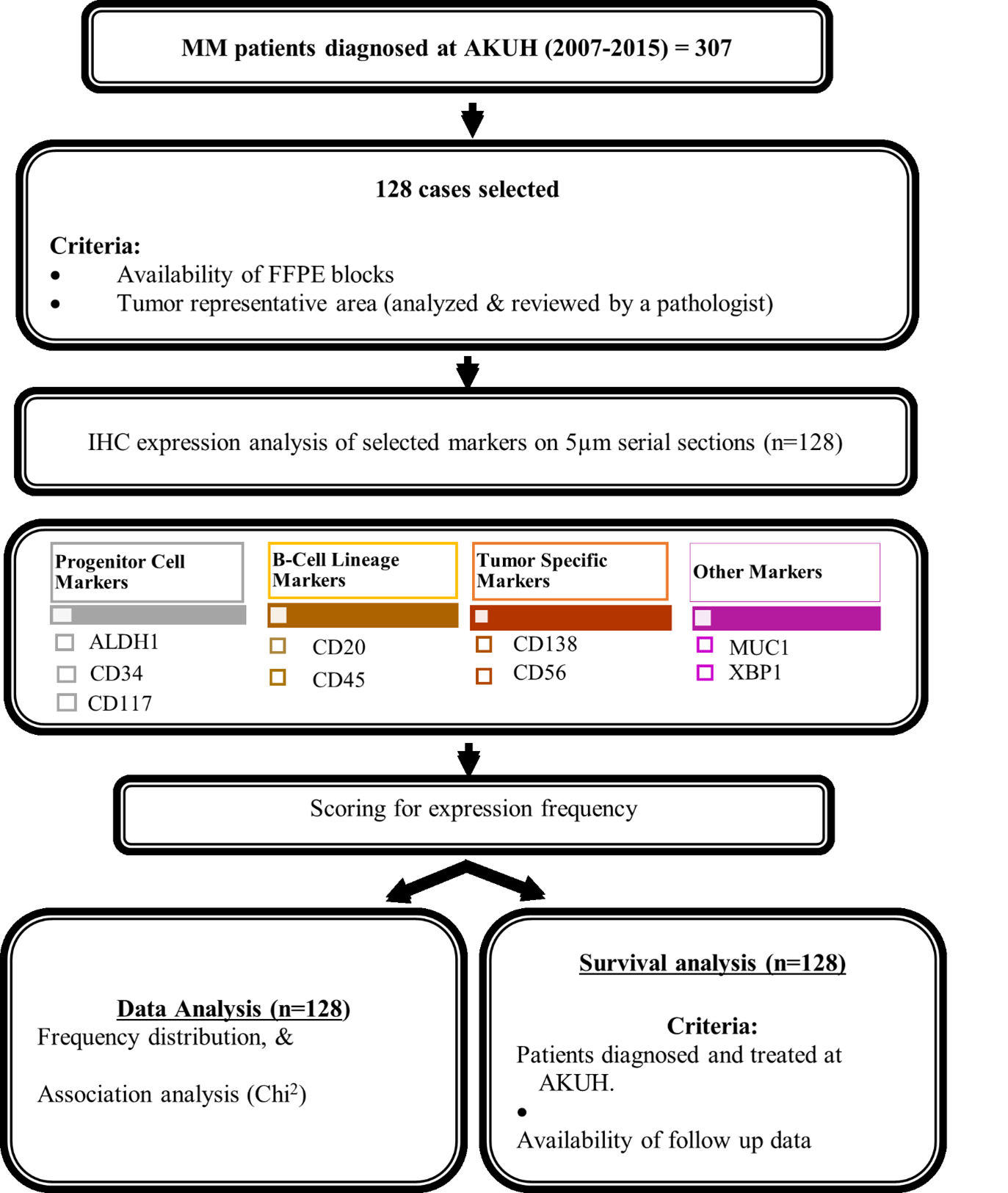

Supplement: S1 Fig — (TIF) [file pone.0320934.s001.tif]
