# Supplementary material for: Prognostic implications of MUC1 and XBP1 concordant expression in multiple myeloma: A retrospective study
Source: PLoS One. 2025 Apr 3;20(4):e0320934. doi: 10.1371/journal.pone.0320934 (PMC11967961; doi:10.1371/journal.pone.0320934)
Supplement: S1 Table — (DOCX) [file pone.0320934.s002.docx]

S1 Table: Panel of antibodies used for immunohistochemical expression in FFPE tissues of myeloma patients.

| Antibody  (Clone) | Source | Catalog ID | Clonality | Host | Antigen Retrieval (pH) | Dilution |
| --- | --- | --- | --- | --- | --- | --- |
| ALDH1 (44/ALDH) | BD Biosciences | 611195 | Monoclonal | Mouse | 6 | 1:100 |
| CD34  (QBEnd10) | DAKO | M7165 | Monoclonal | Mouse | 9 | 1:250 |
| CD20  (L26) | DAKO | M 0755 | Monoclonal | Mouse | 9 | 1:250 |
| CD45  (2B11PD7/2) | DAKO | M0701 | Monoclonal | Mouse | 9 | 1:250 |
| CD56 | DAKO | M7304 | Monoclonal | Mouse | 9 | 1:250 |
| CD138 | DAKO | M 7228 | Monoclonal | Mouse | 9 | 1:100 |
| MUC1 (EMA) | DAKO | M 0613 | Monoclonal | Mouse | 9 | 1:600 |
| XBP1  (M-186) | Santa Cruz | sc-7160 | Polyclonal | Rabbit | 9 | 1:100 |
