# Supplementary material for: Prognostic implications of MUC1 and XBP1 concordant expression in multiple myeloma: A retrospective study
Source: PLoS One. 2025 Apr 3;20(4):e0320934. doi: 10.1371/journal.pone.0320934 (PMC11967961; doi:10.1371/journal.pone.0320934)
Supplement: S2 Table — (DOCX) [file pone.0320934.s003.docx]

**S2 Table: Scoring criteria for IHC expression (84)**

| **Marker** | **Expression pattern in CD138+ Tumor Cells** | **Scoring criteria** |
| --- | --- | --- |
| **ALDH1** | Cytoplasmic | Any proportion of cytoplasmic expression was recorded as positive |
| **CD 117** | Cytoplasmic & Membranous | Any proportion of cytoplasmic and /or membrane expression was recorded as positive |
| **CD 34** | Membranous | Any proportion of membrane expression was recorded as positive |
| **CD138** | Membranous | Positive: ≥30% cytoplasmic expression in plasm cells  Negative: <30% cytoplasmic expression in plasma cells |
| **CD 20** | Membranous | ***Allred Score= PS + IS***  **Proportion Score (PS):**  0=0; 1=1/100; 2=1/10; 3=1/3; 4=2/3; 5= 1  **Intensity Score (IS):**  0= Negative; 1= Weak; 2 = Intermediate; 4= Strong  **For statistical analysis:**  Negative: ≤ 2  Positive: > 2 |
| **CD 45** | Membranous |  |
| **CD 56** | Membranous |  |
| **MUC1** | Membranous & Cytoplasmic |  |
| **XBP1** | Cytoplasmic & Nuclear |  |

1. Fedchenko N, Reifenrath J: **Different approaches for interpretation and reporting of immunohistochemistry analysis results in the bone tissue - a review**. *Diagn Pathol* 2014, **9**:221.
