# Supplementary material for: Prognostic implications of MUC1 and XBP1 concordant expression in multiple myeloma: A retrospective study
Source: PLoS One. 2025 Apr 3;20(4):e0320934. doi: 10.1371/journal.pone.0320934 (PMC11967961; doi:10.1371/journal.pone.0320934)
Supplement: S3 Table — (DOCX) [file pone.0320934.s004.docx]

**S3 Table: Survival analysis for association of expression of biomarkers with overall survival in patients with MM (n=128).**

| **Variable** | **Total N (128)** | **N of Events** | **Mean Survival (Overall)** | | **Confidence Interval (95%)** | | ***p- value*** |
| --- | --- | --- | --- | --- | --- | --- | --- |
|  |  |  |  |  | **Lower** | **Upper** |  |
| **Gender** | | | | | | | **0.52** |
| **Female** | 40 | 11 | 7.21 |  | 5.79 | 8.63 |  |
| **Male** | 88 | 28 | 6.09 |  | 4.85 | 7.33 |  |
| **Age at Diagnosis (Years)** | | | | | | | **<0.001** |
| **<50** | 29 | 5 | 8.27 |  | 6.38 | 10.16 |  |
| **50-64** | 66 | 17 | 6.56 |  | 5.15 | 7.97 |  |
| **>64** | 33 | 17 | 3.76 |  | 2.63 | 4.88 |  |
| **ALDH1** | | | | | | | ***0.53*** |
| Negative | 64 | 20 | 6.39 |  | 4.94 | 7.85 |  |
| Positive | 64 | 19 | 5.42 |  | 4.68 | 6.16 |  |
| **CD117** | | | | | | | ***0.66*** |
| Negative | 66 | 20 | 6.14 |  | 4.82 | 7.46 |  |
| Positive | 62 | 19 | 6 |  | 4.47 | 7.52 |  |
| **CD34** | | | | | | | ***NA*** |
| Negative | 128 | 39 | 6.36 |  | 5.25 | 7.47 |  |
| Positive | 0 | 0 | 0 |  |  |  |  |
| **CD20** | | | | | | | ***0.71*** |
| Negative | 124 | 38 | 6.37 |  | 5.25 | 7.48 |  |
| Positive | 4 | 1 | 4.22 |  | 1.87 | 6.7 |  |
| **CD56** | | | | | | | ***0.24*** |
| Negative | 49 | 17 | 5.87 |  | 4.28 | 7.46 |  |
| Positive | 79 | 22 | 6.55 |  | 5.31 | 7.79 |  |
| **CD45** | | | | | | | ***0.81*** |
| Negative | 114 | 34 | 6.04 |  | 5.01 | 7.06 |  |
| Positive | 14 | 5 | 6.87 |  | 4.13 | 9.61 |  |
| **MUC1-CT2** | | | | | | | ***0.75*** |
| Negative | 33 | 11 | 6.24 |  | 4.53 | 7.95 |  |
| Positive | 95 | 28 | 6.39 |  | 5.1 | 7.69 |  |
| **MUC1** | | | | | | | ***0.22*** |
| Negative | 45 | 12 | 7.05 |  | 5.37 | 8.72 |  |
| Positive | 83 | 27 | 5.15 |  | 4.26 | 6.04 |  |
| **XBP1** | | | | | | | ***0.95*** |
| Negative | 64 | 19 | 6.67 |  | 5.22 | 8.12 |  |
| Positive | 64 | 20 | 5.14 |  | 4.39 | 5.89 |  |
